# Crystallographic and spectroscopic characterization of two 1-phenyl-1*H*-imidazoles: 4-(1*H*-imidazol-1-yl)benzaldehyde and 1-(4-meth­oxy­phen­yl)-1*H*-imidazole

**DOI:** 10.1107/S2056989023005480

**Published:** 2023-06-30

**Authors:** Isobelle F. McClements, Clara R. Wiesler, Joseph M. Tanski

**Affiliations:** a Department of Chemistry, Vassar College, Poughkeepsie, NY 12604, USA; Texas A & M University, USA

**Keywords:** crystal structure, 1-phenyl-1*H*-imidazole derivatives, weak inter­molecular inter­actions, non-centrosymmetric space group

## Abstract

The title compounds, C_10_H_8_N_2_O, (I), and C_10_H_10_N_2_O, (II), are two 1-phenyl-1*H*-imidazole derivatives, which differ in the substituent *para* to the imidazole group on the arene ring, *i.e.* a benzaldehyde, (I), and an anisole, (II). Both mol­ecules pack with different motifs *via* similar weak C—H⋯N/O inter­actions and differ with respect to the angles between the mean planes of the imidazole and arene rings [24.58 (7)° in (I) and 43.67 (4)° in (II)].

## Abstract 2

Two 1-phenyl-1*H*-imidazoles, 4-(1*H*-imidazol-1-yl)benzaldehyde and 1-(4-meth­oxy­phen­yl)-1*H*-imidazole, differ in the substituent *para* to the imidazole group on the arene ring. Both mol­ecules pack with different motifs *via* similar weak C—H⋯N/O inter­actions and differ with respect to the angles between the mean planes of the imidazole and arene rings.

## 1. Chemical context

*N*-Aryl­ated imidazoles are commonly found in the structures of an array of biologically active compounds (Ananthu *et al.*, 2021 ▸). They have a variety of applications in the medicinal chemistry field, such as use in anti­cancer and anti-inflammatory medications and as anti­viral agents (Shalini *et al.*, 2010 ▸). They are also used in agriculture as fungicides, herbicides, and plant-growth regulators (Emel’yanenko *et al.*, 2017 ▸). 4-(1*H*-Imidazol-1-yl)benzaldehyde, (I), may be synthesized in high yield by treating 4-bromo­benzaldehyde with imidazole in an aprotic solvent with the addition of potassium carbonate and a copper(I) catalyst (Xi *et al.*, 2008 ▸). The yellow solid is a common reagent in the synthesis of various targets with anti­fungal and anti­bacterial activity. It has been shown that (I) could be used to synthesize a series of 3-[4-(1*H*-imidazol-1-yl)phen­yl]prop-2-en-1-ones with anti­fungal, antioxidant, and anti­leishmanial activities (Hussain *et al.*, 2009 ▸). Cream-colored 1-(4-meth­oxy­phen­yl)-1*H*-imidazole, (II), and other similar compounds have been found to work as catalysts in the catalytic epoxidation of olefins with moderate to good yields using mild reaction conditions (Schröder *et al.*, 2009 ▸). Compound (II) can be synthesized in a 99% isolated yield by allowing imidazole and 4-iodo­anisole to react in aceto­nitrile in the presence of cesium carbonate and a copper(II) catalyst (Milenković *et al.*, 2019 ▸). 
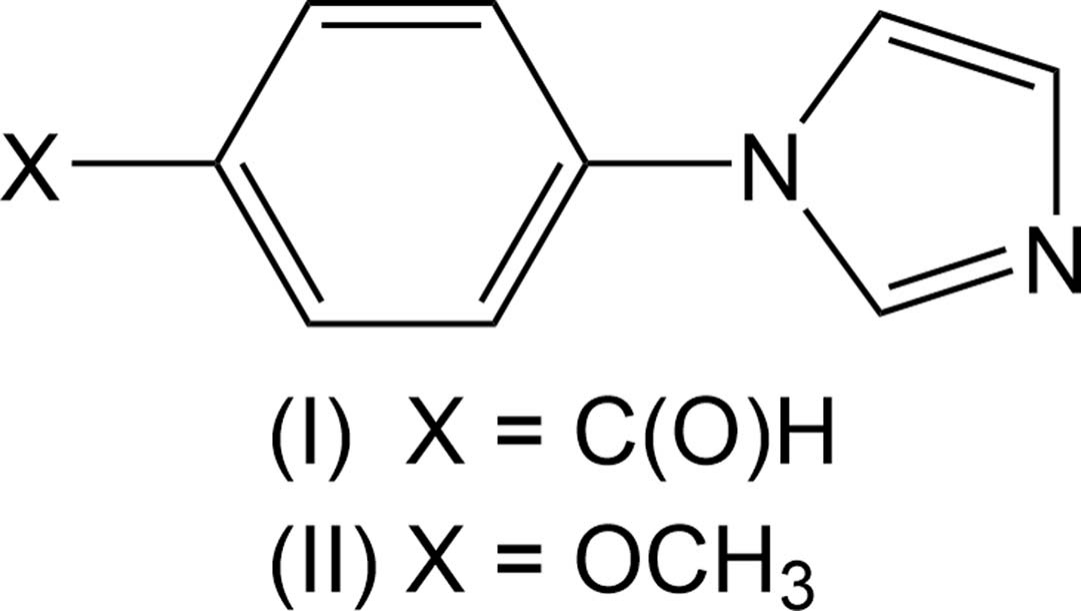


## 2. Structural commentary

**Figure 1:**
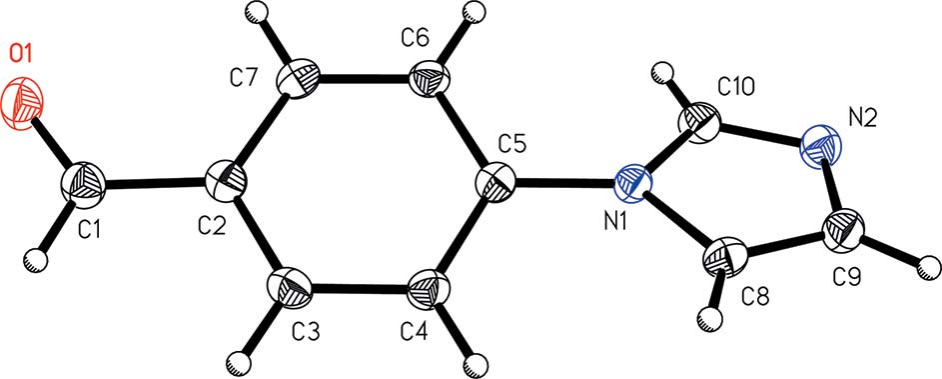
A view of 4-(1*H*-imidazol-1-yl)benzaldehyde, (I), showing the atom-numbering scheme. Displacement ellipsoids are drawn at the 50% probability level.

**Figure 2:**
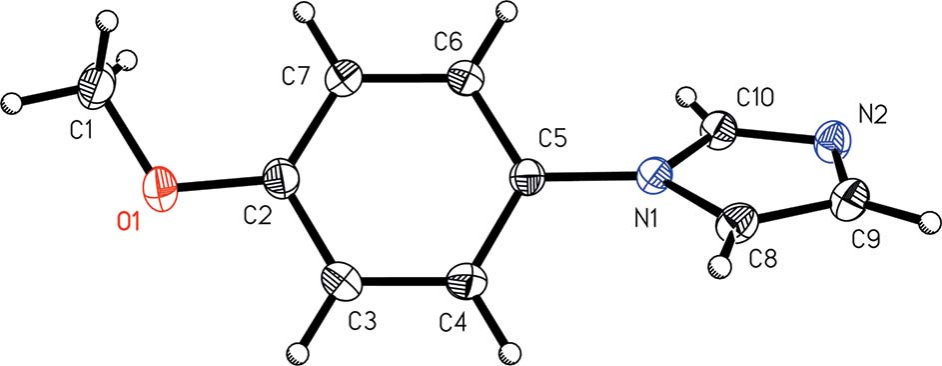
A view of 1-(4-meth­oxy­phen­yl)-1*H*-imidazole, (II), showing the atom-numbering scheme. Displacement ellipsoids are drawn at the 50% probability level.

The mol­ecular structures of the benzaldehyde derivative (I) (Fig. 1 ▸) and the anisole derivative (II) (Fig. 2 ▸) show the *para* nature of the substituent with respect to the imidazole group. The angle between the mean planes of the imidazole and arene rings is 24.58 (7)° in (I) and 43.67 (4)° in (II).

## 3. Supra­molecular features

**Figure 3:**
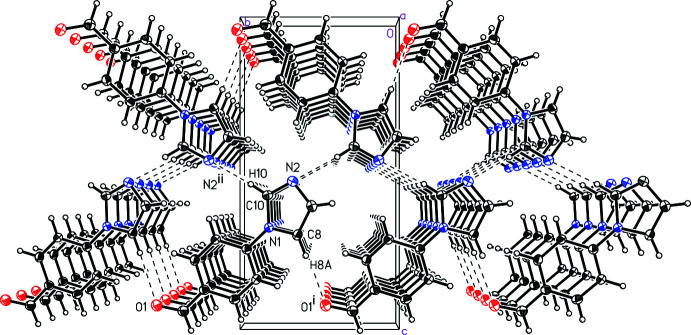
A view of the mol­ecular packing in 4-(1*H*-imidazol-1-yl)benzaldehyde, (I). [Symmetry codes: (i) *x* − 1, *y* − 1, *z*; (ii) −*x* + 1, *y* + 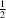 , −*z* + 1.]

The mol­ecules of benzaldehyde derivative (I) are held together in the solid state *via* weak C—H⋯O/N inter­actions (Fig. 3 ▸ and Table 1 ▸). Specifically, imidazole C—H groups inter­act with neighboring benzaldehyde O atoms (C8—H8*A*⋯O1^i^) and imidazole N atoms (C10—H10*A*⋯N2^ii^). The mol­ecules also stack with an offset face-to-face geometrical arrangement of the arene rings, with an inter­molecular centroid-to-centroid distance of 3.7749 (2) Å, a plane-to-centroid distance of 3.5002 (10) Å, and a ring shift of 1.414 (3) Å. Fig. 3 ▸ displays a di-periodic sheet with a thickness roughly equivalent to the length of the *c* axis, where the imidazoles inter­act in the inter­ior and the aldehyde substituents extend to the faces. The sheets then stack in the [001] direction. Notably, (I) crystallizes in the space group *P*2_1_ and is therefore a polar material in the solid state. Polar organic materials formed by achiral mol­ecules are of inter­est in crystal engineering, in particular for nonlinear optical materials (Merritt & Tanski, 2018 ▸).

**Figure 4:**
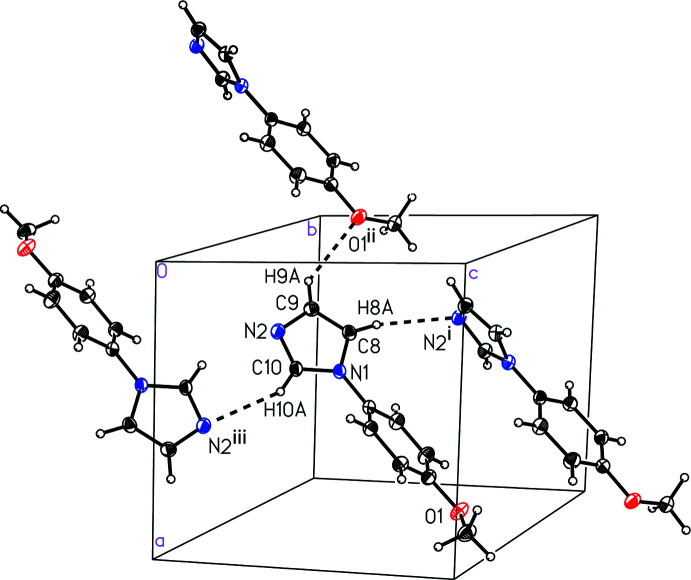
A view of the inter­molecular inter­actions in 1-(4-meth­oxy­phen­yl)-1*H*-imidazole, (II). [Symmetry codes: (i) *x*, −*y* + 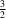 , *z* + 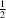 ; (ii) *x* − 1, −*y* + 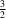 , *z* − 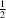 ; (iii) −*x* + 1, −*y* + 1, −*z*.]

**Figure 5:**
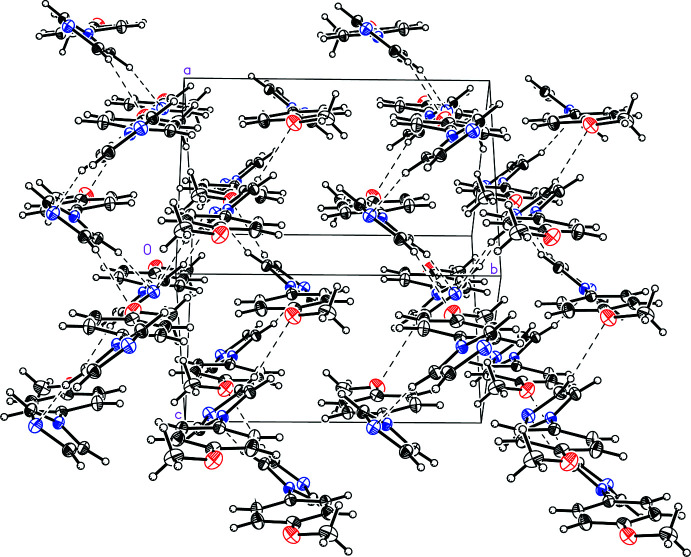
A view of the mol­ecular packing in 1-(4-meth­oxy­phen­yl)-1*H*-imidazole, (II).

Similarly, the mol­ecules of anisole derivative (II) are held together in the solid state *via* weak C—H⋯O/N inter­actions (Fig. 4 ▸ and Table 2 ▸), with the same imidazole C—H groups as (I) inter­acting with a neighboring anisole O atom (C9—H9*A*⋯O1^ii^) and an imidazole N atom (C10—H10*A*⋯N2^iii^). A third weak inter­action links the remaining imidazole H atom with the imidazole N atom (C8—H8*A*⋯N2^i^). Unlike benzaldehyde derivative (I), anisole derivative (II) does not exhibit any π-stacking geometrical arrangement of the arene rings and the mol­ecules pack centrosymmetrically (Fig. 5 ▸).

## 4. Database survey

The Cambridge Structural Database (CSD; Groom *et al.*, 2016 ▸) contains six simple *para*-*X*-substituted 1-phenyl-1*H*-imidazole derivatives: *X* = –NH_2_ (CSD refcode MUFCAS; Liang *et al.*, 2009 ▸), –Br (PAJDUD; Ding *et al.*, 2021 ▸), –I (FIQFUJ; Bejan *et al.*, 2018 ▸), –CO_2_H (IKAWAT; Zheng *et al.*, 2011 ▸), –CO_2_CH_3_ (BEMVUN; Khattri *et al.*, 2016 ▸) and –COCH_3_ (XECDUG; Ibrahim *et al.*, 2012 ▸). The amino and carb­oxy­lic acid derivatives engage in inter­molecular hydrogen bonding with the imidazole N atom and exhibit angles between the mean planes of the imidazole and arene rings of 31.17 (MUFCAS) and 14.51° (IKAWAT). The halide derivatives both contain halide to imidazole nitro­gen inter­molecular con­tacts and angles between the mean planes of the imidazole and arene rings of 35.22 (PAJDUD) and 27.10° (FIQFUJ). Similar to the title compounds (I) and (II), the methyl ester and methyl ketone derivatives pack *via* weak C—H⋯N/O inter­actions and with angles between the mean planes of the imidazole and arene rings of 24.83 (BEMVUN) and 1.04° (XECDUG). In XECDUG, a mol­ecule of water hydrogen bonds to the 1*H*-imidazole H and *ortho*-phenyl H of a neighboring mol­ecule, holding the planes of the imidazole and arene rings nearly coplanar. Inspection of the bond lengths of the imidazole ring for all eight derivatives reveals that they are remarkably similar.

## 5. Synthesis and crystallization

4-(1*H*-Imidazol-1-yl)benzaldehyde (98%), (I), and 1-(4-meth­oxy­phen­yl)-1*H*-imidazole (98%), (II), were purchased from Aldrich Chemical Company, USA, and were used as received.

## 6. Refinement

Crystal data, data collection and structure refinement details are summarized in Table 3 ▸. H atoms on C atoms were included in calculated positions and refined using a riding model, with C—H = 0.95 Å and *U*_iso_(H) = 1.2*U*_eq_(C) for aryl H atoms, and C—H = 0.98 Å and *U*_iso_(H) = 1.5*U*_eq_(C) for methyl H atoms.

## 7. Analytical data

### 7.1. 4-(1*H*-Imidazol-1-yl)benzaldehyde, (I)

^1^H NMR (Bruker Avance III HD 400 MHz, CDCl_3_): δ 7.26 (*m*, 1H, C_imid_ *H*), 7.39 (*m*, 1H, C_imid_ *H*), 7.60 (*d*, 2H, C_ar­yl_ *H*, *J* = 8.6 Hz), 7.99 (*s*, 1H, C_imid_ *H*), 8.03 (*d*, 2H, C_ar­yl_ *H*, *J* = 8.6 Hz), 10.05 [*s*, 1H, C(O)*H*]. ^13^C NMR (^13^C{^1^H}, 100.6 MHz, CDCl_3_): δ 117.54 (*C*_imid_H), 120.97 (*C*_ar­yl_H), 131.23 (*C*_imid_H), 131.48 (*C*_ar­yl_H), 134.84 (*C*_ar­yl_), 135.28 (*C*_imid_H), 141.60 (*C*_ar­yl_), 190.48 [*C*(O)H]. IR (Thermo Nicolet iS50, ATR, cm^−1^): 3138 (w, C_ar­yl_—H str), 3109 (*m*, C_ar­yl_—H str), 2818 and 2746 (*m*, =C—H aldehyde Fermi doublet str), 1676 (*s*, C=O str), 1604 (*s*, arom. C=C str), 1519 (*s*, arom. C=C str), 1481 (*s*, arom. C=C str), 1439 (*m*), 1400 (*s*), 1375 (*s*), 1310 (*s*), 1268 (*s*), 1220 (*s*), 1171 (*s*), 1120 (*m*), 1105 (*m*), 1059 (*s*), 971 (*m*), 959 (*s*), 902 (*w*), 830 (*s*), 752 (*s*), 692 (*m*), 752 (*s*), 692 (*m*), 648 (*s*), 617 (*m*), 530 (*m*), 513 (*s*), 447 (*m*), 413 (*m*). GC–MS (Agilent Technologies 7890A GC/5975C MS): *M* ^+^ = 172 amu.

### 7.2. 1-(4-Meth­oxy­phen­yl)-1*H*-imidazole, (II)

^1^H NMR (Bruker Avance III HD 400 MHz, CDCl_3_): δ 3.85 (*s*, 3H, OC*H*_3_), 6.98 (*d*, 2H, C_ar­yl_ *H*, *J* = 8.9 Hz), 7.20 (*m*, 2H, C_imid_ *H*), 7.30 (*d*, 2H, C_ar­yl_ *H*, *J* = 8.9 Hz), 7.78 (*m*, 1H, C_imid_ *H*). ^13^C NMR (^13^C{^1^H}, 100.6 MHz, CDCl_3_): δ 55.56 (O*C*H_3_), 114.87 (*C*_ar­yl_H), 118.83 (*C*_imid_H), 123.19 (*C*_ar­yl_H), 129.97 (*C*_ar­yl_), 130.68 (*C*_imid_H), 135.89 (*C*_imid_H), 158.92 (*C*_ar­yl_). IR (Thermo Nicolet iS50, ATR, cm^−1^): 3128 (*m*, C_ar­yl_—H str), 3107 (*m*, C_ar­yl_—H str), 2961 (*w*, C_alk­yl_—H str), 2918 (*w*, C_alk­yl_—H str), 2838 (*m*, C_alk­yl_—H str), 2052 (*w*), 1877 (*w*), 1634 (*w*), 1610 (*m*), 1591 (*w*), 1517 (*s*, arom. C=C str), 1471 (*s*, arom. C=C str), 1459 (*m*), 1447 (*w*), 1332 (*m*), 1321 (*s*), 1302 (*m*), 1267 (*s*), 1256 (*s*), 1241 (*s*), 1192 (*s*), 1173 (*m*), 1109 (*s*), 1100 (*s*), 1061 (*s*), 1029 (*s*), 961 (*m*), 910 (*m*), 873 (*w*), 840 (*s*), 823 (*s*), 798 (*s*), 780 (*s*), 762 (*s*), 664 (*s*), 649 (*s*), 614 (*m*), 539 (*s*), 490 (*m*), 434 (*w*). GC–MS (Agilent Technologies 7890A GC/5975C MS): *M* ^+^ = 174 amu.

## Supplementary Material

Crystal structure: contains datablock(s) global, I, II. DOI: 10.1107/S2056989023005480/jy2032sup1.cif

Structure factors: contains datablock(s) I. DOI: 10.1107/S2056989023005480/jy2032Isup2.hkl

Structure factors: contains datablock(s) II. DOI: 10.1107/S2056989023005480/jy2032IIsup3.hkl

Supporting information file. DOI: 10.1107/S2056989023005480/jy2032Isup4.cml

Supporting information file. DOI: 10.1107/S2056989023005480/jy2032IIsup5.cml

CCDC references: 2267421, 2267419

Additional supporting information:  crystallographic information; 3D view; checkCIF report

## Figures and Tables

**Table 1:** Hydrogen-bond geometry (Å, °) for (I)

*D*—H⋯*A*	*D*—H	H⋯*A*	*D*⋯*A*	*D*—H⋯*A*
C8—H8*A*⋯O1^i^	0.95	2.51	3.458 (2)	176
C10—H10*A*⋯N2^ii^	0.95	2.51	3.449 (2)	173

Symmetry codes: (i) 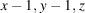 ; (ii) 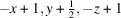 .

**Table 2:** Hydrogen-bond geometry (Å, °) for (II)

*D*—H⋯*A*	*D*—H	H⋯*A*	*D*⋯*A*	*D*—H⋯*A*
C8—H8*A*⋯N2^i^	0.95	2.55	3.4391 (11)	157
C9—H9*A*⋯O1^ii^	0.95	2.56	3.3048 (11)	136
C10—H10*A*⋯N2^iii^	0.95	2.52	3.3004 (11)	140

Symmetry codes: (i) 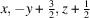 ; (ii) 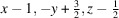 ; (iii) 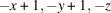 .

**Table 3:** Experimental details Experiments were carried out at 125 K using a Bruker APEXII CCD diffractometer. Absorption was corrected for by multi-scan methods (*SADABS*; Bruker, 2016 ▸). Refinement was on 119 parameters. H-atom parameters were constrained.

	(I)	(II)
Crystal data		
Chemical formula	C_10_H_8_N_2_O	C_10_H_10_N_2_O
*M*_r_	172.18	174.20
Crystal system, space group	Monoclinic, *P*2_1_	Monoclinic, *P*2_1_/*c*
*a*, *b*, *c* (Å)	3.7749 (2), 7.3711 (5), 14.4524 (9)	8.5663 (12), 11.2143 (16), 9.1635 (13)
β (°)	91.096 (2)	94.448 (2)
*V* (Å^3^)	402.07 (4)	877.6 (2)
*Z*	2	4
Radiation type	Cu *K*α	Mo *K*α
μ (mm^−1^)	0.77	0.09
Crystal size (mm)	0.37 × 0.20 × 0.05	0.40 × 0.25 × 0.15

Data collection		
*T*_min_, *T*_max_	0.80, 0.96	0.92, 0.99
No. of measured, independent and observed [*I* > 2σ(*I*)] reflections	5673, 1482, 1466	21397, 2678, 2332
*R*_int_	0.029	0.031
(sin θ/λ)_max_ (Å^−1^)	0.615	0.715

Refinement		
*R*[*F* ^2^ > 2σ(*F* ^2^)], *wR*(*F* ^2^), *S*	0.027, 0.079, 1.14	0.040, 0.119, 1.04
No. of reflections	1482	2678
No. of restraints	1	0
Δρ_max_, Δρ_min_ (e Å^−3^)	0.19, −0.15	0.35, −0.29
Absolute structure	Flack *x* determined using 652 quotients [(*I* ^+^) − (*I* ^−^)]/[(*I* ^+^) + (*I* ^−^)] (Parsons *et al.*, 2013 ▸); Hooft *y* = 0.11(6) calculated with *OLEX2* (Dolomanov *et al.*, 2009 ▸)	–
Absolute structure parameter	0.09 (7)	–

Computer programs: *APEX3* and *SAINT* (Bruker, 2013 ▸), *SHELXT2018* (Sheldrick, 2015*a* ▸), *SHELXL2017* (Sheldrick, 2015*b* ▸), *SHELXTL2014* (Sheldrick, 2008 ▸), *OLEX2* (Dolomanov *et al.*, 2009 ▸), and *Mercury* (Macrae *et al.*, 2020 ▸).

## supplementary crystallographic information

### 4-(1H-Imidazol-1-yl)benzaldehyde (I) Crystal data

**Table:**

C_10_H_8_N_2_O	*F*(000) = 180
*M**_r_* = 172.18	*D*_x_ = 1.422 Mg m^−^^3^
Monoclinic, *P*2_1_	Cu *K*α radiation, λ = 1.54178 Å
*a* = 3.7749 (2) Å	Cell parameters from 5426 reflections
*b* = 7.3711 (5) Å	θ = 3.1–71.6°
*c* = 14.4524 (9) Å	µ = 0.77 mm^−^^1^
β = 91.096 (2)°	*T* = 125 K
*V* = 402.07 (4) Å^3^	Plate, clear colourless
*Z* = 2	0.37 × 0.20 × 0.05 mm

### 4-(1H-Imidazol-1-yl)benzaldehyde (I) Data collection

**Table:**

Bruker APEXII CCD diffractometer	1482 independent reflections
Radiation source: Cu IuS micro-focus source	1466 reflections with *I* > 2σ(*I*)
Detector resolution: 8.3333 pixels mm^-1^	*R*_int_ = 0.029
φ and ω scans	θ_max_ = 71.6°, θ_min_ = 3.1°
Absorption correction: multi-scan (SADABS; Bruker, 2016)	*h* = −4→4
*T*_min_ = 0.80, *T*_max_ = 0.96	*k* = −8→7
5673 measured reflections	*l* = −17→16

### 4-(1H-Imidazol-1-yl)benzaldehyde (I) Refinement

**Table:**

Refinement on *F*^2^	Hydrogen site location: inferred from neighbouring sites
Least-squares matrix: full	H-atom parameters constrained
*R*[*F*^2^ > 2σ(*F*^2^)] = 0.027	*w* = 1/[σ^2^(*F*_o_^2^) + (0.0495*P*)^2^ + 0.0471*P*] where *P* = (*F*_o_^2^ + 2*F*_c_^2^)/3
*wR*(*F*^2^) = 0.079	(Δ/σ)_max_ < 0.001
*S* = 1.14	Δρ_max_ = 0.19 e Å^−^^3^
1482 reflections	Δρ_min_ = −0.15 e Å^−^^3^
119 parameters	Extinction correction: SHELXL2017 (Sheldrick, 2015b)
1 restraint	Extinction coefficient: 0.021 (6)
Primary atom site location: dual	Absolute structure: Flack *x* determined using 652 quotients [(I+)-(I-)]/[(I+)+(I-)] (Parsons *et al.*, 2013); Hooft *y* = 0.11(6) calculated with OLEX2 (Dolomanov *et al.*, 2009).
Secondary atom site location: difference Fourier map	Absolute structure parameter: 0.09 (7)

### 4-(1H-Imidazol-1-yl)benzaldehyde (I) Special details

**Table:**

Geometry. All esds (except the esd in the dihedral angle between two l.s. planes) are estimated using the full covariance matrix. The cell esds are taken into account individually in the estimation of esds in distances, angles and torsion angles; correlations between esds in cell parameters are only used when they are defined by crystal symmetry. An approximate (isotropic) treatment of cell esds is used for estimating esds involving l.s. planes.

### 4-(1H-Imidazol-1-yl)benzaldehyde (I) Fractional atomic coordinates and isotropic or equivalent isotropic displacement parameters (Å^2^)

**Table:**

	*x*	*y*	*z*	*U*_iso_*/*U*_eq_	
O1	0.8970 (4)	0.9617 (2)	0.09534 (10)	0.0289 (4)	
N1	0.3041 (4)	0.2861 (2)	0.33233 (10)	0.0166 (4)	
N2	0.2148 (4)	0.1490 (2)	0.46723 (11)	0.0222 (4)	
C1	0.8366 (5)	0.8021 (3)	0.07934 (13)	0.0230 (4)	
H1A	0.889378	0.758391	0.019326	0.028*	
C2	0.6890 (5)	0.6719 (3)	0.14516 (12)	0.0185 (4)	
C3	0.6418 (5)	0.4920 (3)	0.11753 (13)	0.0203 (4)	
H3A	0.698927	0.4571	0.056322	0.024*	
C4	0.5120 (5)	0.3631 (3)	0.17851 (12)	0.0192 (4)	
H4A	0.480518	0.240754	0.159434	0.023*	
C5	0.4286 (4)	0.4165 (3)	0.26824 (12)	0.0169 (4)	
C6	0.4704 (4)	0.5971 (3)	0.29652 (12)	0.0183 (4)	
H6A	0.409116	0.632564	0.357352	0.022*	
C7	0.6015 (5)	0.7236 (3)	0.23531 (13)	0.0199 (4)	
H7A	0.632366	0.846022	0.254394	0.024*	
C8	0.1451 (4)	0.1207 (3)	0.31214 (12)	0.0191 (4)	
H8A	0.086412	0.073743	0.25258	0.023*	
C9	0.0906 (5)	0.0395 (3)	0.39529 (13)	0.0212 (4)	
H9A	−0.017271	−0.075833	0.403072	0.025*	
C10	0.3399 (5)	0.2943 (3)	0.42704 (13)	0.0204 (4)	
H10A	0.443164	0.393702	0.45957	0.025*	

### 4-(1H-Imidazol-1-yl)benzaldehyde (I) Atomic displacement parameters (Å^2^)

**Table:**

	*U*^11^	*U*^22^	*U*^33^	*U*^12^	*U*^13^	*U*^23^
O1	0.0395 (9)	0.0225 (9)	0.0249 (7)	−0.0053 (6)	0.0026 (6)	0.0022 (6)
N1	0.0195 (7)	0.0148 (9)	0.0154 (7)	0.0001 (6)	−0.0004 (5)	0.0002 (6)
N2	0.0268 (8)	0.0210 (10)	0.0188 (7)	−0.0002 (6)	0.0009 (6)	0.0014 (6)
C1	0.0245 (9)	0.0236 (12)	0.0209 (9)	−0.0008 (8)	−0.0004 (7)	0.0000 (8)
C2	0.0179 (8)	0.0201 (11)	0.0175 (8)	0.0005 (7)	−0.0013 (7)	0.0002 (7)
C3	0.0212 (9)	0.0230 (11)	0.0166 (9)	0.0020 (7)	0.0012 (7)	−0.0018 (7)
C4	0.0230 (9)	0.0166 (11)	0.0180 (8)	−0.0005 (7)	−0.0001 (7)	−0.0021 (7)
C5	0.0151 (8)	0.0176 (10)	0.0180 (8)	0.0009 (7)	−0.0018 (6)	0.0007 (7)
C6	0.0212 (8)	0.0175 (10)	0.0164 (8)	0.0015 (7)	0.0006 (7)	−0.0021 (7)
C7	0.0213 (9)	0.0162 (10)	0.0221 (10)	0.0002 (7)	−0.0015 (7)	−0.0017 (7)
C8	0.0202 (8)	0.0168 (10)	0.0203 (8)	−0.0005 (7)	−0.0009 (6)	−0.0027 (7)
C9	0.0219 (9)	0.0187 (11)	0.0229 (9)	0.0004 (7)	0.0007 (7)	0.0016 (7)
C10	0.0243 (9)	0.0207 (11)	0.0163 (9)	0.0005 (7)	−0.0013 (7)	−0.0012 (7)

### 4-(1H-Imidazol-1-yl)benzaldehyde (I) Geometric parameters (Å, º)

**Table:**

O1—C1	1.219 (3)	C3—H3A	0.9500
N1—C10	1.374 (2)	C4—C5	1.397 (2)
N1—C8	1.388 (2)	C4—H4A	0.9500
N1—C5	1.421 (2)	C5—C6	1.401 (3)
N2—C10	1.311 (2)	C6—C7	1.383 (3)
N2—C9	1.391 (2)	C6—H6A	0.9500
C1—C2	1.469 (2)	C7—H7A	0.9500
C1—H1A	0.9500	C8—C9	1.362 (3)
C2—C3	1.395 (3)	C8—H8A	0.9500
C2—C7	1.403 (2)	C9—H9A	0.9500
C3—C4	1.391 (3)	C10—H10A	0.9500
			
C10—N1—C8	106.39 (15)	C4—C5—N1	119.84 (17)
C10—N1—C5	126.28 (15)	C6—C5—N1	119.32 (16)
C8—N1—C5	127.19 (15)	C7—C6—C5	119.59 (17)
C10—N2—C9	105.21 (15)	C7—C6—H6A	120.2
O1—C1—C2	125.41 (17)	C5—C6—H6A	120.2
O1—C1—H1A	117.3	C6—C7—C2	120.28 (18)
C2—C1—H1A	117.3	C6—C7—H7A	119.9
C3—C2—C7	119.54 (17)	C2—C7—H7A	119.9
C3—C2—C1	118.92 (15)	C9—C8—N1	105.83 (16)
C7—C2—C1	121.53 (17)	C9—C8—H8A	127.1
C4—C3—C2	120.81 (16)	N1—C8—H8A	127.1
C4—C3—H3A	119.6	C8—C9—N2	110.49 (18)
C2—C3—H3A	119.6	C8—C9—H9A	124.8
C3—C4—C5	118.95 (17)	N2—C9—H9A	124.8
C3—C4—H4A	120.5	N2—C10—N1	112.07 (16)
C5—C4—H4A	120.5	N2—C10—H10A	124.0
C4—C5—C6	120.84 (16)	N1—C10—H10A	124.0
			
O1—C1—C2—C3	−178.55 (18)	N1—C5—C6—C7	178.08 (15)
O1—C1—C2—C7	0.2 (3)	C5—C6—C7—C2	0.6 (2)
C7—C2—C3—C4	−0.6 (3)	C3—C2—C7—C6	0.2 (3)
C1—C2—C3—C4	178.21 (16)	C1—C2—C7—C6	−178.52 (16)
C2—C3—C4—C5	0.1 (3)	C10—N1—C8—C9	0.62 (19)
C3—C4—C5—C6	0.7 (3)	C5—N1—C8—C9	176.67 (16)
C3—C4—C5—N1	−178.42 (15)	N1—C8—C9—N2	−0.6 (2)
C10—N1—C5—C4	153.02 (17)	C10—N2—C9—C8	0.4 (2)
C8—N1—C5—C4	−22.3 (2)	C9—N2—C10—N1	0.0 (2)
C10—N1—C5—C6	−26.2 (3)	C8—N1—C10—N2	−0.4 (2)
C8—N1—C5—C6	158.54 (16)	C5—N1—C10—N2	−176.52 (16)
C4—C5—C6—C7	−1.1 (2)		

### 4-(1H-Imidazol-1-yl)benzaldehyde (I) Hydrogen-bond geometry (Å, º)

**Table:**

*D*—H···*A*	*D*—H	H···*A*	*D*···*A*	*D*—H···*A*
C8—H8*A*···O1^i^	0.95	2.51	3.458 (2)	176
C10—H10*A*···N2^ii^	0.95	2.51	3.449 (2)	173

Symmetry codes: (i) *x*−1, *y*−1, *z*; (ii) −*x*+1, *y*+1/2, −*z*+1.

### 1-(4-Methoxyphenyl)-1H-imidazole (II) Crystal data

**Table:**

C_10_H_10_N_2_O	*F*(000) = 368
*M**_r_* = 174.20	*D*_x_ = 1.318 Mg m^−^^3^
Monoclinic, *P*2_1_/*c*	Mo *K*α radiation, λ = 0.71073 Å
*a* = 8.5663 (12) Å	Cell parameters from 8588 reflections
*b* = 11.2143 (16) Å	θ = 2.4–30.4°
*c* = 9.1635 (13) Å	µ = 0.09 mm^−^^1^
β = 94.448 (2)°	*T* = 125 K
*V* = 877.6 (2) Å^3^	Plate, colourless
*Z* = 4	0.40 × 0.25 × 0.15 mm

### 1-(4-Methoxyphenyl)-1H-imidazole (II) Data collection

**Table:**

Bruker APEXII CCD diffractometer	2678 independent reflections
Radiation source: sealed X-ray tube, Bruker APEXII CCD	2332 reflections with *I* > 2σ(*I*)
Graphite monochromator	*R*_int_ = 0.031
Detector resolution: 8.3333 pixels mm^-1^	θ_max_ = 30.6°, θ_min_ = 2.4°
φ and ω scans	*h* = −12→12
Absorption correction: multi-scan (SADABS; Bruker, 2016)	*k* = −16→15
*T*_min_ = 0.92, *T*_max_ = 0.99	*l* = −13→13
21397 measured reflections	

### 1-(4-Methoxyphenyl)-1H-imidazole (II) Refinement

**Table:**

Refinement on *F*^2^	Primary atom site location: dual
Least-squares matrix: full	Secondary atom site location: difference Fourier map
*R*[*F*^2^ > 2σ(*F*^2^)] = 0.040	Hydrogen site location: inferred from neighbouring sites
*wR*(*F*^2^) = 0.119	H-atom parameters constrained
*S* = 1.04	*w* = 1/[σ^2^(*F*_o_^2^) + (0.0687*P*)^2^ + 0.1966*P*] where *P* = (*F*_o_^2^ + 2*F*_c_^2^)/3
2678 reflections	(Δ/σ)_max_ < 0.001
119 parameters	Δρ_max_ = 0.35 e Å^−^^3^
0 restraints	Δρ_min_ = −0.29 e Å^−^^3^

### 1-(4-Methoxyphenyl)-1H-imidazole (II) Special details

**Table:**

Geometry. All esds (except the esd in the dihedral angle between two l.s. planes) are estimated using the full covariance matrix. The cell esds are taken into account individually in the estimation of esds in distances, angles and torsion angles; correlations between esds in cell parameters are only used when they are defined by crystal symmetry. An approximate (isotropic) treatment of cell esds is used for estimating esds involving l.s. planes.

### 1-(4-Methoxyphenyl)-1H-imidazole (II) Fractional atomic coordinates and isotropic or equivalent isotropic displacement parameters (Å^2^)

**Table:**

	*x*	*y*	*z*	*U*_iso_*/*U*_eq_	
N1	0.50111 (8)	0.64826 (6)	0.27442 (7)	0.01696 (16)	
N2	0.35189 (9)	0.60120 (7)	0.07295 (8)	0.02110 (17)	
O1	0.98420 (8)	0.63969 (6)	0.71768 (7)	0.02769 (18)	
C1	1.02429 (11)	0.53203 (10)	0.79493 (10)	0.0289 (2)	
H1A	1.108753	0.547969	0.87077	0.043*	
H1B	1.059244	0.472373	0.726426	0.043*	
H1C	0.932403	0.501862	0.840608	0.043*	
C2	0.86571 (10)	0.63397 (8)	0.60922 (9)	0.02084 (18)	
C3	0.81994 (11)	0.74301 (8)	0.54587 (10)	0.02479 (19)	
H3A	0.870761	0.814315	0.579384	0.03*	
C4	0.70105 (11)	0.74809 (8)	0.43457 (9)	0.02198 (18)	
H4A	0.670793	0.822403	0.391376	0.026*	
C5	0.62626 (10)	0.64337 (7)	0.38651 (9)	0.01708 (17)	
C6	0.67205 (10)	0.53468 (7)	0.44827 (9)	0.01901 (17)	
H6A	0.621215	0.463503	0.414318	0.023*	
C7	0.79220 (10)	0.52919 (8)	0.55986 (9)	0.02071 (18)	
H7A	0.823565	0.454653	0.601777	0.025*	
C8	0.37965 (10)	0.72974 (7)	0.26290 (9)	0.01978 (18)	
H8A	0.362414	0.793692	0.327719	0.024*	
C9	0.28976 (10)	0.69928 (8)	0.13921 (9)	0.02107 (18)	
H9A	0.197198	0.739844	0.10349	0.025*	
C10	0.47846 (10)	0.57358 (7)	0.15758 (9)	0.01911 (18)	
H10A	0.545959	0.509019	0.139087	0.023*	

### 1-(4-Methoxyphenyl)-1H-imidazole (II) Atomic displacement parameters (Å^2^)

**Table:**

	*U*^11^	*U*^22^	*U*^33^	*U*^12^	*U*^13^	*U*^23^
N1	0.0198 (3)	0.0156 (3)	0.0153 (3)	0.0015 (2)	0.0003 (2)	−0.0014 (2)
N2	0.0226 (4)	0.0219 (4)	0.0183 (3)	0.0020 (3)	−0.0011 (3)	−0.0016 (3)
O1	0.0249 (3)	0.0327 (4)	0.0239 (3)	−0.0069 (3)	−0.0077 (3)	0.0039 (3)
C1	0.0249 (4)	0.0389 (5)	0.0222 (4)	−0.0024 (4)	−0.0028 (3)	0.0082 (4)
C2	0.0194 (4)	0.0251 (4)	0.0178 (4)	−0.0035 (3)	0.0003 (3)	0.0010 (3)
C3	0.0271 (4)	0.0204 (4)	0.0260 (4)	−0.0055 (3)	−0.0037 (3)	−0.0010 (3)
C4	0.0264 (4)	0.0162 (4)	0.0228 (4)	−0.0015 (3)	−0.0013 (3)	0.0004 (3)
C5	0.0188 (4)	0.0176 (4)	0.0148 (3)	−0.0004 (3)	0.0009 (3)	−0.0006 (3)
C6	0.0205 (4)	0.0167 (4)	0.0195 (4)	−0.0014 (3)	−0.0001 (3)	−0.0001 (3)
C7	0.0214 (4)	0.0205 (4)	0.0200 (4)	−0.0013 (3)	−0.0001 (3)	0.0031 (3)
C8	0.0231 (4)	0.0166 (4)	0.0197 (4)	0.0034 (3)	0.0024 (3)	−0.0010 (3)
C9	0.0211 (4)	0.0209 (4)	0.0211 (4)	0.0037 (3)	0.0005 (3)	0.0011 (3)
C10	0.0221 (4)	0.0181 (4)	0.0170 (3)	0.0018 (3)	0.0004 (3)	−0.0031 (3)

### 1-(4-Methoxyphenyl)-1H-imidazole (II) Geometric parameters (Å, º)

**Table:**

N1—C10	1.3612 (10)	C3—C4	1.3854 (12)
N1—C8	1.3827 (10)	C3—H3A	0.9500
N1—C5	1.4271 (10)	C4—C5	1.3928 (11)
N2—C10	1.3200 (10)	C4—H4A	0.9500
N2—C9	1.3828 (11)	C5—C6	1.3876 (11)
O1—C2	1.3658 (10)	C6—C7	1.3947 (11)
O1—C1	1.4279 (12)	C6—H6A	0.9500
C1—H1A	0.9800	C7—H7A	0.9500
C1—H1B	0.9800	C8—C9	1.3634 (11)
C1—H1C	0.9800	C8—H8A	0.9500
C2—C7	1.3920 (12)	C9—H9A	0.9500
C2—C3	1.3967 (12)	C10—H10A	0.9500
			
C10—N1—C8	106.62 (7)	C5—C4—H4A	120.3
C10—N1—C5	126.57 (7)	C6—C5—C4	120.21 (8)
C8—N1—C5	126.81 (7)	C6—C5—N1	120.01 (7)
C10—N2—C9	104.78 (7)	C4—C5—N1	119.78 (7)
C2—O1—C1	117.23 (7)	C5—C6—C7	120.46 (7)
O1—C1—H1A	109.5	C5—C6—H6A	119.8
O1—C1—H1B	109.5	C7—C6—H6A	119.8
H1A—C1—H1B	109.5	C2—C7—C6	119.38 (8)
O1—C1—H1C	109.5	C2—C7—H7A	120.3
H1A—C1—H1C	109.5	C6—C7—H7A	120.3
H1B—C1—H1C	109.5	C9—C8—N1	105.73 (7)
O1—C2—C7	124.61 (8)	C9—C8—H8A	127.1
O1—C2—C3	115.48 (8)	N1—C8—H8A	127.1
C7—C2—C3	119.91 (8)	C8—C9—N2	110.64 (7)
C4—C3—C2	120.57 (8)	C8—C9—H9A	124.7
C4—C3—H3A	119.7	N2—C9—H9A	124.7
C2—C3—H3A	119.7	N2—C10—N1	112.23 (7)
C3—C4—C5	119.47 (8)	N2—C10—H10A	123.9
C3—C4—H4A	120.3	N1—C10—H10A	123.9
			
C1—O1—C2—C7	−6.43 (13)	N1—C5—C6—C7	−178.75 (7)
C1—O1—C2—C3	174.10 (8)	O1—C2—C7—C6	179.88 (8)
O1—C2—C3—C4	179.87 (8)	C3—C2—C7—C6	−0.68 (13)
C7—C2—C3—C4	0.38 (14)	C5—C6—C7—C2	0.19 (13)
C2—C3—C4—C5	0.41 (14)	C10—N1—C8—C9	0.16 (9)
C3—C4—C5—C6	−0.91 (13)	C5—N1—C8—C9	179.98 (7)
C3—C4—C5—N1	178.46 (7)	N1—C8—C9—N2	−0.15 (10)
C10—N1—C5—C6	−44.15 (12)	C10—N2—C9—C8	0.07 (10)
C8—N1—C5—C6	136.07 (9)	C9—N2—C10—N1	0.03 (10)
C10—N1—C5—C4	136.49 (9)	C8—N1—C10—N2	−0.12 (10)
C8—N1—C5—C4	−43.30 (12)	C5—N1—C10—N2	−179.94 (7)
C4—C5—C6—C7	0.61 (13)		

### 1-(4-Methoxyphenyl)-1H-imidazole (II) Hydrogen-bond geometry (Å, º)

**Table:**

*D*—H···*A*	*D*—H	H···*A*	*D*···*A*	*D*—H···*A*
C8—H8*A*···N2^i^	0.95	2.55	3.4391 (11)	157
C9—H9*A*···O1^ii^	0.95	2.56	3.3048 (11)	136
C10—H10*A*···N2^iii^	0.95	2.52	3.3004 (11)	140

Symmetry codes: (i) *x*, −*y*+3/2, *z*+1/2; (ii) *x*−1, −*y*+3/2, *z*−1/2; (iii) −*x*+1, −*y*+1, −*z*.
